# Environmental concentrations of copper and iron can alter the evolution of antimicrobial resistance of *E. coli* against ciprofloxacin and doxycycline

**DOI:** 10.3389/fmicb.2025.1539807

**Published:** 2025-03-05

**Authors:** Indorica Sutradhar, Prinjali Kalyan, Kelechi Chukwu, Akebe Luther King Abia, Joshua Mbanga, Sabiha Essack, Davidson H. Hamer, Muhammad H. Zaman

**Affiliations:** ^1^Department of Biomedical Engineering, Boston University, Boston, MA, United States; ^2^Antimicrobial Research Unit, School of Health Sciences, University of KwaZulu-Natal, Durban, South Africa; ^3^Environmental Research Foundation, Westville, South Africa; ^4^Department of Global Health, School of Public Health, Boston University, Boston, MA, United States; ^5^Section of Infectious Diseases, Department of Medicine, Boston University Chobanian & Avedisian School of Medicine, Boston, MA, United States; ^6^Center on Emerging Infectious Diseases Research, Boston University, Boston, MA, United States; ^7^Center for Forced Displacement, Boston University, Boston, MA, United States; ^8^Howard Hughes Medical Institute, Boston University, Boston, MA, United States

**Keywords:** antimicrobial resistance, wastewater, trace metal pollution, antibiotic pollution, computational modeling

## Abstract

Antimicrobial resistance (AMR) is a global health challenge and there is increasing recognition of the role of the environment, particularly wastewater, in the development and spread of AMR. Although trace metals are common contaminants in wastewater, the quantitative effects of trace metals on AMR in wastewater settings remain understudied. We experimentally determined the interactions between common antibiotic residues and metal ions found in wastewater and investigated their effects on the development of antibiotic resistance in *Escherichia coli* over time. These data were then used to expand on a previously developed computational model of antibiotic resistance development in continuous flow settings to incorporate the effects of trace metals acting in combination with multiple antibiotic residues. We found that copper and iron, interact with both ciprofloxacin and doxycycline at wastewater relevant concentrations. This can significantly affect resistance development due to antibiotic chelation of the metal ions causing a reduction in the antibiotics’ bioactivity. Furthermore, modeling the effect of these interactions in wastewater systems showed the potential for metal ions in wastewater to significantly increase the development of antibiotic resistant *E. coli* populations. These results demonstrate the need to quantitatively understand the effects of trace metal-antibiotic interactions on AMR development in wastewater.

## 1 Introduction

Antimicrobial resistance (AMR) is a pressing global health challenge that has directly resulted in 1.27 million fatalities in 2019 alone, an annual mortality rate expected to increase to 1.91 million/year by 2050 (Antimicrobial Resistance Collaborators, 2022). While global efforts in addressing AMR have largely focused on human health and agriculture sectors, there is increasing evidence that the environment also plays a key role in the development and spread of AMR (Antimicrobial Resistance Collaborators, 2022; Nadimpalli et al., 2020; Bengtsson-Palme and Larsson, 2016). One of the major reservoirs of AMR in the environment is wastewater, where effluent from hospital, pharmaceutical, domestic, and agricultural sources can contain antibiotic residues and/or bacteria containing antibiotic resistance genes (ARGs) (Antimicrobial Resistance Collaborators, 2022; Nadimpalli et al., 2020; Bengtsson-Palme and Larsson, 2016; Sutradhar et al., 2021; Muñoz-Aguayo et al., 2007; Fang et al., 2023; Kraupner et al., 2021; Kurasam et al., 2022; Hubeny et al., 2021). While the quantitative effects of these antibiotic residues on the development of AMR in wastewater have begun to be studied (Muñoz-Aguayo et al., 2007; Fang et al., 2023; Kraupner et al., 2021; Kurasam et al., 2022) and computationally modeled (Sutradhar et al., 2021), there still exists a gap in modeling the effects of other components of the complex wastewater environment and their effects on AMR development.

One of the most common contaminants in wastewater are trace metal ions, and their presence can have significant environmental and human health implications (Hubeny et al., 2021). Industrial and domestic activities can lead to the discharge of metals such as zinc, copper, and iron into wastewater (Hubeny et al., 2021; Imai et al., 2020). For instance, studies in Pakistan and Ghana showed iron concentrations in wastewater ranging from 0.4 to 4.9 mg/L (Akhtar et al., 2022; Abagale et al., 2013). Furthermore, the presence of these heavy metals in wastewater has been increasing in recent years due to industrial growth including the plating and electroplating industry, batteries, pesticides, the mining industries, the textile industry, as well as the metal smelting, and petrochemicals industries (Qasem et al., 2021). Additionally, these metals do not biodegrade and can pose long term threats to environmental and human health (Hubeny et al., 2021; Qasem et al., 2021). These metals commonly found in wastewater can have a significant impact on the development of AMR in the environment (Hubeny et al., 2021; Zhang et al., 2018; Faure et al., 2021; Kara et al., 1991). Studies have shown that trace metals, including copper and zinc, at environmentally relevant and sub-inhibitory concentrations, can promote horizontal gene transfer (HGT) of ARGs between *Escherichia coli* strains (Hubeny et al., 2021; Zhang et al., 2018). Additionally, some trace metals can interact with antibiotic residues forming complexes with changed bioactivity (Faure et al., 2021; Kara et al., 1991; Ghandour et al., 1992; Albert and Rees, 1956; Imaoka et al., 2014; Vos et al., 2020). For example, tetracyclines are known chelators of metals including iron and copper (Ghandour et al., 1992; Albert and Rees, 1956). These metal ions can interfere with the binding of tetracycline to the 30S bacterial ribosome and iron chelation by doxycycline has been seen to decrease the anti-pseudomonal activity of doxycycline (Faure et al., 2021). Similarly, studies have found that ciprofloxacin can bind to ferric ion which interacts with the 4-keto and 3-carboxyl groups on ciprofloxacin; this binding in turn reduces ciprofloxacin bioavailability (Kara et al., 1991). Studies on trace metal-antibiotic interactions and AMR have found that the presence of zinc alongside ciprofloxacin can select against ciprofloxacin-resistant bacteria (Vos et al., 2020). However, the quantitative effects of trace metals on *de novo* AMR development in wastewater settings have not been adequately studied. There is thus a need to incorporate trace metals into studies of AMR development in the environment. Our study attempted to fill this gap by experimentally determining the degree of interaction between common antibiotic residues and metal ions seen in wastewater and probing the effect of these interactions on the development of antibiotic resistance over time. Furthermore, we use these results to expand our previously developed computational model of antibiotic resistance development in continuous flow settings to include the effects of trace metals present alongside antibiotic residues on the development of AMR in wastewater (Sutradhar et al., 2021).

## 2 Methods

### 2.1 MIC determination

Wild type *E. coli* MG1655 cultured at 37°C in LB media was grown in 96-well plates with 2-fold concentration increments across the plate of the antibiotic of interest (ciprofloxacin, doxycycline, erythromycin, streptomycin, or rifampin). The highest concentration of each antibiotic was as follows: ciprofloxacin: 4.8 mg/L; doxycycline: 384 mg/L; erythromycin: 16.384 g/L; streptomycin: 256 mg/L; rifampin: 3.2 g/L. Broth volumes in each well were kept static at 100 μL and each media condition was run in biological triplicate, started from unique overnight cultures (*n* = 3). Minimum inhibitory concentration (MIC) was determined to be at the lowest concentration of antibiotic where no growth was observed visually and verified via OD500 reading in microplate reader.

### 2.2 Metal-antibiotic interaction determination

Wild type *E. coli* MG1655 was cultured for 24 h at 37°C in LB media supplemented with 2-fold concentration increments of ciprofloxacin, doxycycline, erythromycin, streptomycin, and rifampin in 96-well plates. The highest concentration of each antibiotic was as follows: ciprofloxacin: 4.8 mg/L; doxycycline: 384 mg/L; erythromycin: 16.384 g/L; streptomycin: 256 mg/L; rifampin: 3.2 g/L. Various increasing concentrations of copper (II) sulfate pentahydrate (Sigma-Aldrich, St. Louis, USA) or iron (III) sulfate hydrate (Sigma-Aldrich, St. Louis, USA) prepared in sterile water, chosen for their common occurrence in wastewater and their known interactions with antibiotics, were added to each antibiotic condition (Hubeny et al., 2021; Zhang et al., 2018; Faure et al., 2021; Kara et al., 1991). Broth volumes in each well were kept static at 100 μL and each combination of metal and antibiotic was run in biological triplicate. The concentrations of metals surveyed were between 0.5 and 100 mg/L, a range determined to be relevant to wastewater environments (Akhtar et al., 2022; Abagale et al., 2013). The concentration of metal required to double the MIC of ciprofloxacin or doxycycline for *E. coli* as compared to antibiotic not supplemented with metal ion was determined according to the above MIC protocol.

### 2.3 Serial passaging

*E. coli* MG1655 (ATCC 700926) cultured at 37°C in LB media was grown in 96-well plates with 2-fold increments of ciprofloxacin with the maximum concentration at 4.8 mg/L. Additionally, the metal of interest (0.5 mg/L or 5 mg/L copper or 5 mg/L or 10 mg/L iron) was added to each well. Broth volumes in each well were kept static at 100 μL and each combination of metal and antibiotic was run in biological triplicate (*n* = 3). We selected the bacteria in the well closest to 50% of the inhibitory concentration (IC50) to seed new bacterial cultures (bacterial load of 10^5^ cfu/mL) on the same dose series of ciprofloxacin at ~24 h, for 10 days. Bacteria serially passaged in media with antibiotic or metal concentrations for the duration of the experiments served as the control groups. This procedure was repeated with 2-fold increments of doxycycline with the maximum concentration at 384 mg/L and 5 mg/L copper and iron and two-fold increments of streptomycin with the maximum concentration at 256 mg/L and 0.05 mg/L, 0.5 mg/L and 5 mg/L copper. Changes in resistance of the cultures were measured through daily MIC measurements in response to each antibiotic without the presence of metal ions, determined according to the above MIC protocol.

### 2.4 Statistical analysis

For the serial passaging experiments, statistical significance between conditions was determined to be at a *p*-value of less than 0.05 using a two-tailed Student’s t-test performed on the MIC concentration values on day 10 of each condition which had been performed in biological triplicate (*n* = 3), compared to the control condition where no metal was present.

### 2.5 Computational model

The model used in this paper is based on a previously developed and validated model of the growth of antibiotic resistant bacterial populations in continuous flow environments (Sutradhar et al., 2021; Sutradhar et al., 2023). This model was built on prior studies and was extended to incorporate a variety of wastewater relevant inputs which can be broadly classified into bacterial parameters, environmental parameters, and antibiotic parameters. Bacteria specific input factors include the growth rates of antibiotic susceptible and resistant strains and mutation rates in response to subinhibitory concentrations of antibiotic. The antibiotic specific inputs, such as bactericidal activity, allow for the study of the effects of antibiotic pollution on the development of resistance. Additionally, environmental inputs, including physical inflow and outflow rates and antibiotic residue concentrations, allow for the modeling of resistance development in a variety of settings of interest. Ordinary differential equations incorporating these input parameters were used to model an output of resistant bacterial populations over time, thus allowing for the prediction of resistant population development (Table 1). The model includes two primary equations that describe the concentrations of two antibiotics (*C_1_* and *C_2_*) over time, as well as equations that track the susceptible population (*S*), populations resistant to each antibiotic through chromosomal mutations (*R_1_* and *R_2_*), and a population that has developed resistance to both antibiotics through mutation (*R_m_*) (Table 1). For each antibiotic, the model accounts for both the environmental concentration (*E*) and its rate of clearance (*k_e_*). The growth of bacterial populations is modeled through a growth rate parameter (*α*), which is constrained by an experimentally determined carrying capacity (*N_max_*). The antibiotics’ bioactivity is captured in the bacterial killing term by the killing rate coefficients ( $\delta_{\max,1}$ and $\delta_{\max,2}$ ), and IC50 values where bacterial growth inhibition from antibiotic is at 50%—either for susceptible strains ( $C_{S}^{50}$ ) or for resistant strains ( $C_{R,1}^{50},\;C_{R,2}^{50}$ ). The model also incorporates factors for bacterial influx ( $g_{s}$ , $g_{Rm}$ , $g_{R1},g_{R2},g_{Rp}$ ) and outflow (*k_T_*) rates (Table 2), which depend on the physical flow rates in the system being studied, in this case the flow parameters set in the eVOLVER system used for experimental validation (Vos et al., 2020). Mutation due to antibiotic pressure is modeled through concentration-dependent mutation rates (*m(C)*). In this work, we expanded the model to include the effects of two metal ions, copper and iron, on resistance development in response to various antibiotic combinations. This was done by adjusting the effective concentration of each antibiotic by the degree to which the antibiotic chelated the metal ion concentration that was present (Equations 1–6). Each antibiotic is now modeled as an “available antibiotic concentration” (*C_1_* and C*_2_*). These concentrations are modeled with experimentally determined terms for the antibiotic residue concentration in the environment (*E*), the antibiotic clearance rate (*k_e_*), and chelation of two metal ions (*M_1_* and *M_2_*). The degree of chelation (*k_M_*) term is approximated for each antibiotic-metal pair by calculating fractional amount of antibiotic that can be bound to the given metal ion from the results of the metal-antibiotic interaction determination described above (1/concentration required to double MIC).

(1) $$\frac{dC_{1}}{dt}=E_{1}-k_{e}C_{1}-k_{M1C1}M_{1}C_{1}-k_{M2C1}M_{2}C_{1}$$

(2) $$\frac{dC_{2}}{dt}=E_{2}-k_{e}C_{2}-k_{M1C2}M_{1}C_{2}-k_{M2C2}M_{2}C_{2}$$

(3) $$\begin{array}{l} \frac{dS}{dt}=\alpha_{S}\left(1-\frac{R_{m}+R_{1}+R_{2}+S}{N_{\text{max}}}\right)S+g_{s}-k_{T}S\\ \;-syn*\delta_{\max,1}\left(\frac{C_{1}}{C_{1}+C_{S}^{50}}\right)S-syn*\delta_{\max,2}\left(\frac{C_{2}}{C_{2}+C_{S}^{50}}\right)S \end{array}$$

(4) $$\begin{array}{l} \frac{dR_{m}}{dt}=\alpha_{R}\left(1-\frac{R_{m}+R_{1}+R_{2}+S}{N_{\text{max}}}\right)R_{m}+g_{Rm}\\ \;-k_{T}R_{m}-syn*\delta_{\max,1}\left(\frac{C_{1}}{C_{1}+C_{R,1}^{50}}\right)R_{m}\\ \;-syn*\delta_{\max,2}\left(\frac{C_{2}}{C_{2}+C_{R,2}^{50}}\right)R_{m}+syn*m_{T}\left(C_{1},C_{2}\right)S\\ \;+R_{1}m_{2}\left(C_{2}\right)+R_{2}m_{1}\left(C_{1}\right) \end{array}$$

(5) $$\begin{array}{l} \frac{dR_{1}}{dt}=\alpha_{R,1}\left(1-\frac{R_{m}+R_{1}+R_{2}+S}{N_{\text{max}}}\right)R_{1}+g_{R1}-k_{T}R_{1}\\ -syn*\delta_{\max,1}\left(\frac{C_{1}}{C_{1}+C_{R,1}^{50}}\right)R_{1}-syn*\delta_{\max,2}\left(\frac{C_{2}}{C_{2}+C_{S}^{50}}\right)\\ R_{1}+m_{1}\left(C_{1}\right)S \end{array}$$

(6) $$\begin{array}{l} \frac{dR_{2}}{dt}=\alpha_{R,2}\left(1-\frac{R_{m}+R_{1}+R_{2}+S}{N_{\text{max}}}\right)R_{2}+g_{R2}-k_{T}R_{2}\\ -syn*\delta_{\max,1}\left(\frac{C_{1}}{C_{1}+C_{S}^{50}}\right)R_{2}-syn*\delta_{\max,2}\left(\frac{C_{2}}{C_{2}+C_{R,2}^{50}}\right)\\ R_{2}+m_{2}\left(C_{2}\right)S \end{array}$$

**Table 1:** Model variables, definitions, and initial values, adapted from Sutradhar et al. (2021).

Variable	Definitions	Initial value
*C_1_*	Effective Antibiotic 1 Concentration (μg/mL)	0
*C_2_*	Effective Antibiotic 2 Concentration (μg/mL)	0
*S*	Susceptible (cells/mL)	3*10^8^
*R_m_*	Resistant to both Antibiotic 1 and Antibiotic 2 from Chromosomal Mutation (cells/mL)	10
*R_1_*	Resistant to only Antibiotic 1 from Chromosomal Mutation (cells/mL)	100
*R_2_*	Resistant to only Antibiotic 2 from Chromosomal Mutation (cells/mL)	100

**Table 2:** Model parameter definitions and values, adapted from Sutradhar et al. (2021).

Parameter	Definition	Value	
E	Antibiotic Load ((μg/mL)/h)	Load of Interest	
M	Metal Ion Load ((μg/mL)/h)	Load of Interest	
*syn*	Synergy Parameter (non-dimensional)	Experimentally determined by FIC	
$k_{e}$	Antibiotic Clearance Rate (1/h)	1.97	
$k_{M}$	Chelation of Metal Ion (mL/μg)	Dox and Iron	0.2
		Dox and Copper	0.1
		Cip and Iron	0.1
		Cip and Copper	0.05
$\alpha_{S}$	Growth Rate of Susceptible Bacteria (1/h)	13.66	
$\alpha_{R}$	Growth Rate of Bacteria Resistant from Mutation (1/h)	Dox	10.75
		Cip	6.1
$N_{\text{max}}$	Carrying Capacity (cells/mL)	10^9^	
$g_{s}$ , $g_{Rm}$ , $g_{R1},g_{R2},g_{Rp}$	Bacterial Influx Rates ((cells/mL)/h)	0	
$k_{T}$	Bacterial Efflux Rate (1/h)	0.8	
$\delta_{\max,1}$ , $\delta_{\max,2}$	Bacterial Killing Rate in Response to Antibiotic 1 and Antibiotic 2 (1/h)	1.97	
$C_{S}^{50},C_{R,1}^{50},\;C_{R,2}^{50}$	IC50 (μg/mL)	Half MIC Value of Antibiotic	
$m_{1}\left(C_{1}\right)$ , $m_{2}\left(C_{2}\right)$	Mutation Frequency under Antibiotic 1 and Antibiotic 2 (1/h)	Dox	8.35*10^−3^*( $\frac{C_{1}}{2C_{S}^{50}}$ )
		Cip	8.35*10^−5^*( $\frac{C_{2}}{2C_{S}^{50}}$ )

*Eq Set 1*. Model equations for available antibiotic concentrations in the presence of two metal ion concentrations, adapted from Sutradhar et al. (2021).

## 3 Results

Initial experiments examining the interactions between various antibiotics and iron and copper solutions demonstrated that only doxycycline and ciprofloxacin showed changes in antibiotic activity against *E. coli* in the presence of the metal ions (Table 3). Erythromycin, streptomycin and rifampicin showed no change in MIC at any environmentally relevant concentration of either iron or copper (up to 100 mg/L of metal ion as determined by values reported in literature). Furthermore, metal alone had no measurable antimicrobial activity in the tested range and no effect on MIC evolution (Supplementary Data). No variance was observed in any condition. Beyond the concentrations of iron and copper observed to double MIC of *E. coli* to ciprofloxacin and doxycycline, only doxycycline showed continued increase in MIC up to a four-fold increase at 50 mg/L of iron. However, doxycycline maintained two-fold increases in MIC up to 100 mg/L Cu and ciprofloxacin maintained two-fold increases in MIC for both 100 mg/L Fe and 100 mg/L Cu.

**Table 3:** Interactions of various antibiotics with iron and copper solutions (*n* = 3).

Antibiotic	Concentration of iron to double MIC	Concentration of copper to double MIC
Doxycycline	5 mg/L	20 mg/L
Ciprofloxacin	20 mg/L	50 mg/L
Erythromycin	No Interaction	No Interaction
Streptomycin	No Interaction	No Interaction
Rifampicin	No Interaction	No Interaction

**Figure 1:**
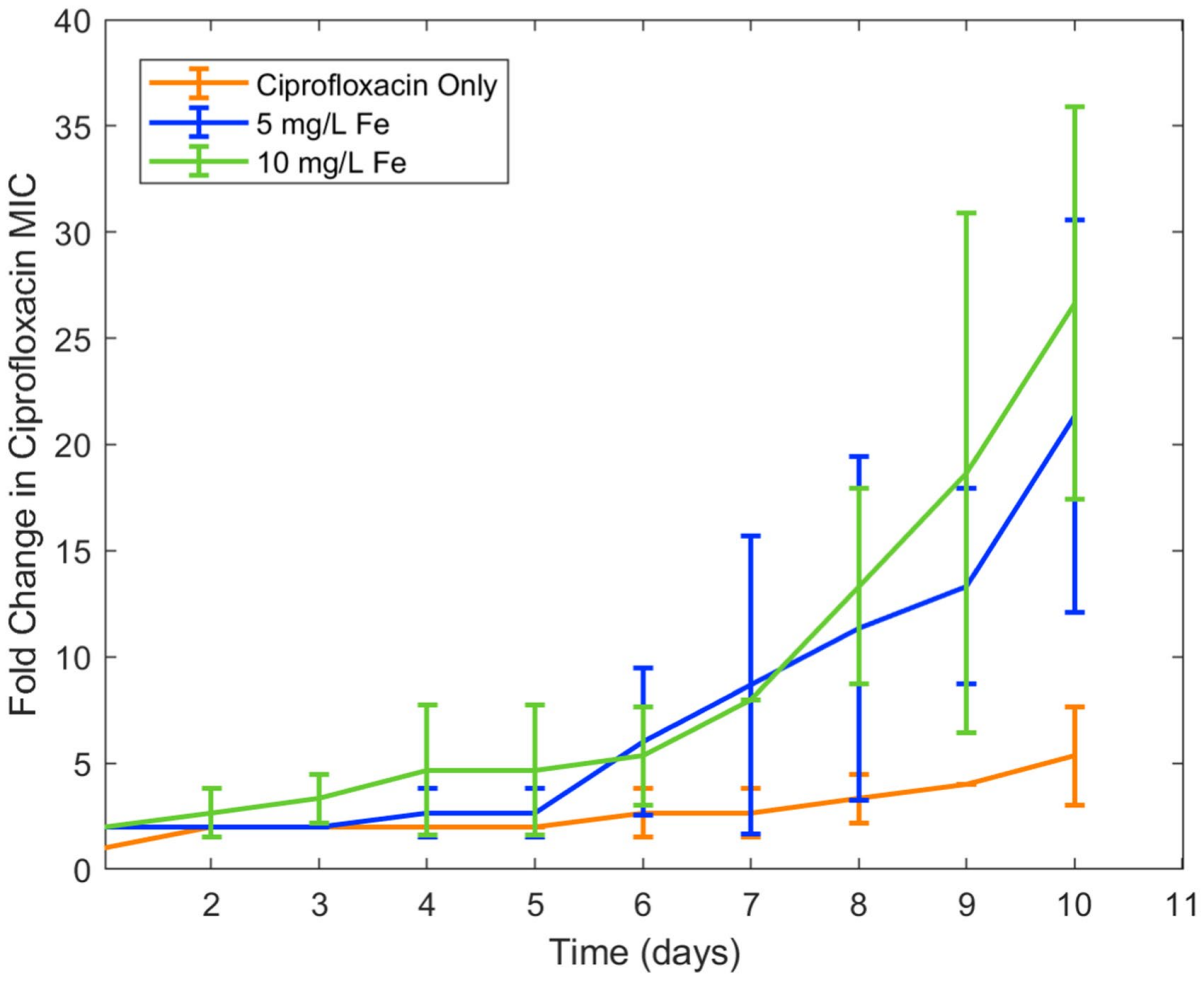
Effect of iron on ciprofloxacin resistance development. Error bars show standard deviation (*n* = 3).

This reduction of bioactivity from chelation was of particular interest because prior literature has demonstrated the effect of reducing concentration of antibiotic on resistance development (Ching and Zaman, 2020; Weinstein and Zaman, 2018). Due to their change in activity in response to iron and copper at environmentally relevant concentrations as well as its high relevance in clinical settings in low- and middle-income countries, ciprofloxacin and doxycycline were chosen for longitudinal studies of the effect of these metal ions on resistance development over time. In the first of these experiments, the effect of iron ions alongside ciprofloxacin significantly increased the development of ciprofloxacin resistance in *E. coli* (Figure 1). While standard deviation appears high in this and the following studies, this is due to the experimental design where antibiotic concentrations are increased exponentially.

**Figure 2:**
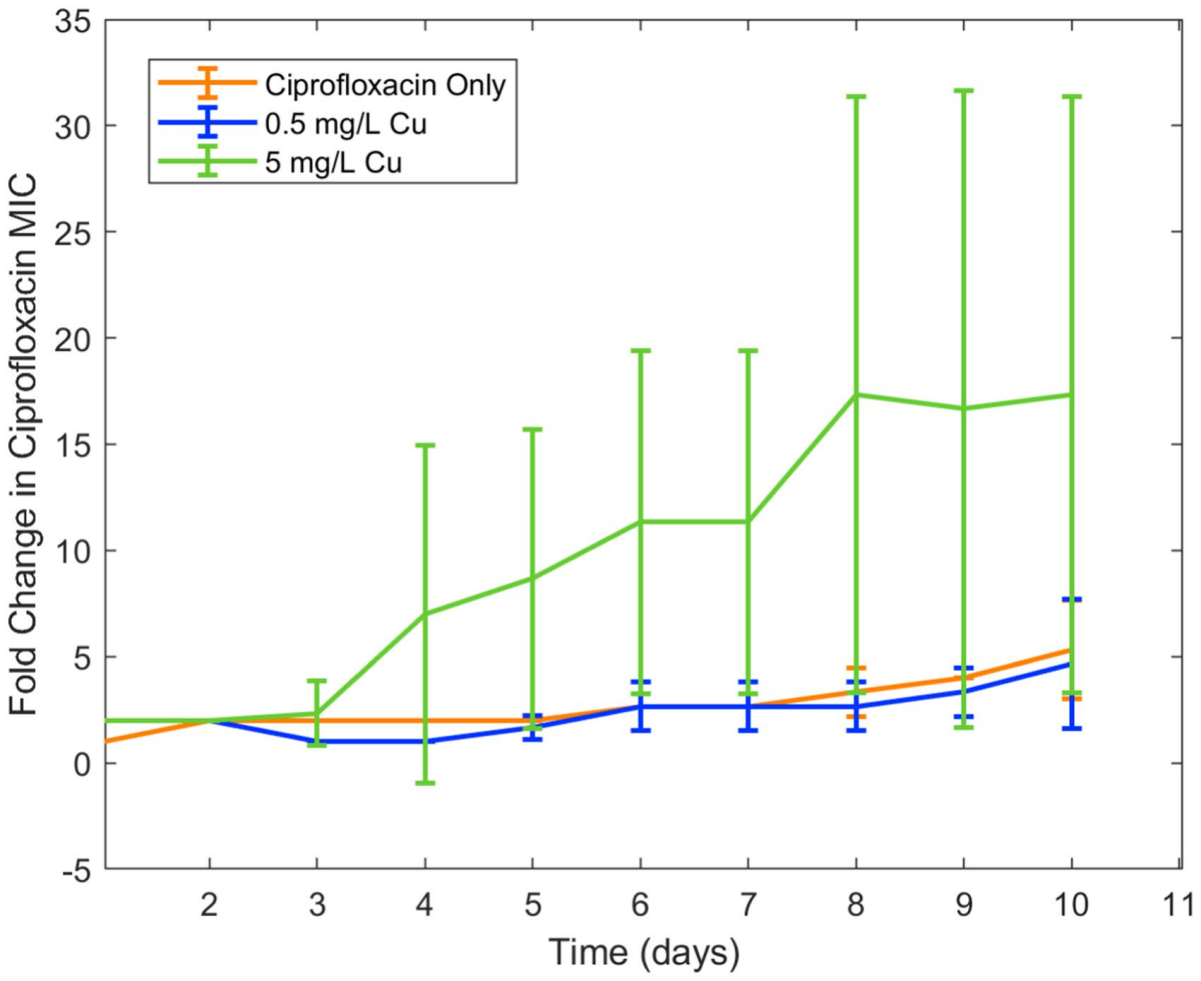
Effect of copper on ciprofloxacin resistance development. Error bars show standard deviation (*n* = 3).

**Figure 3:**
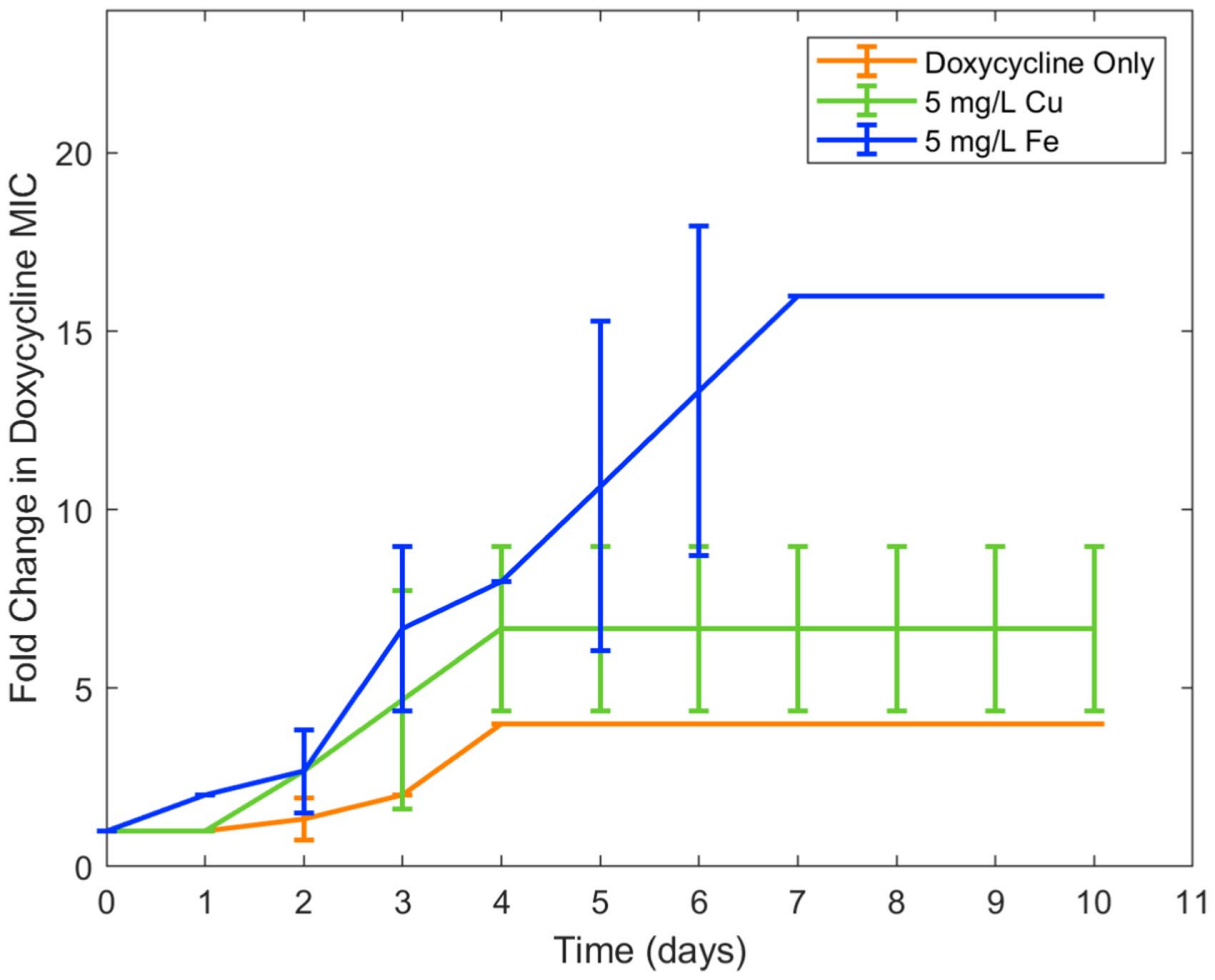
Effect of copper and iron on doxycycline resistance development. Error bars show standard deviation (*n* = 3).

**Figure 4:**
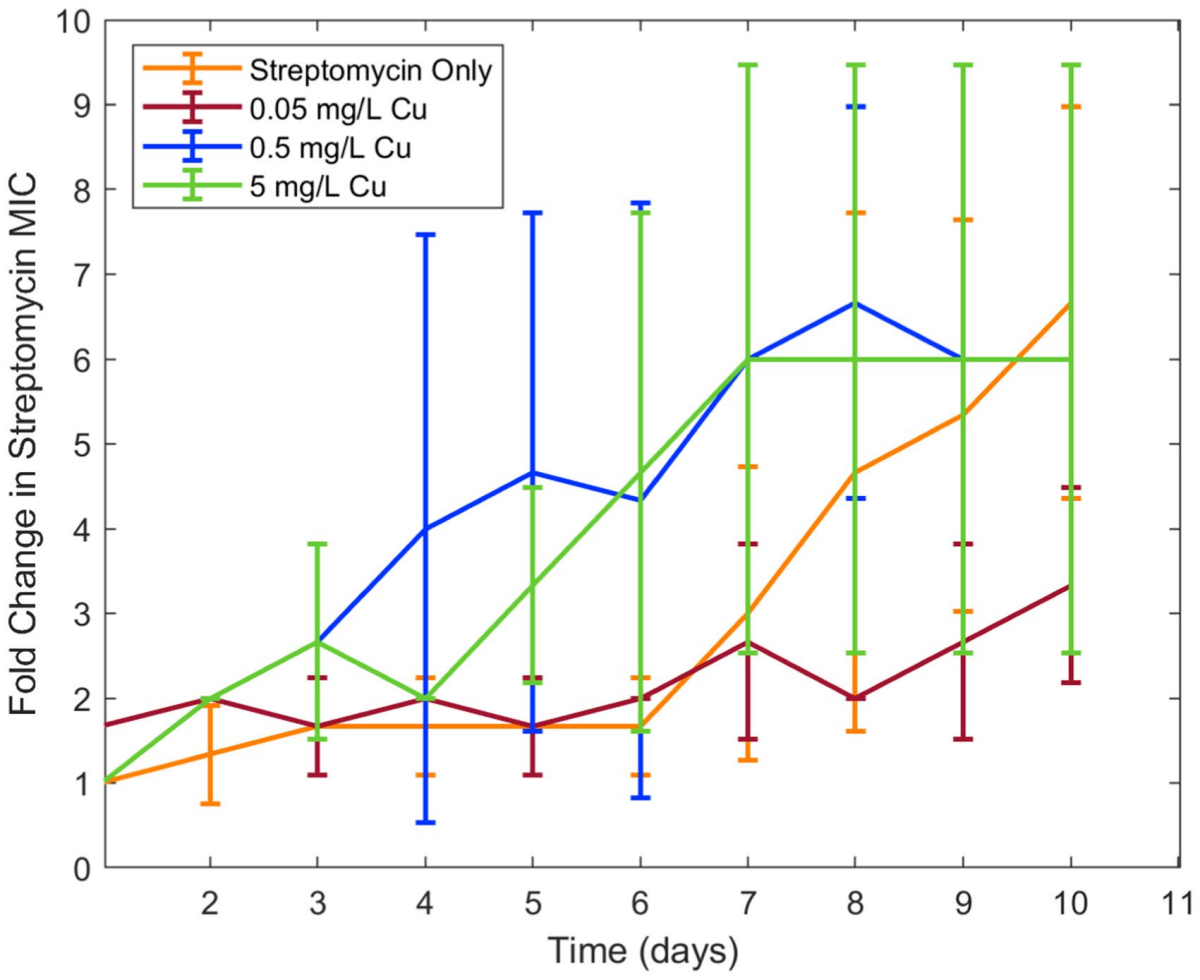
Effect of copper on streptomycin resistance development. Error bars show standard deviation (*n* = 3).

Because copper was known to have similar binding to ciprofloxacin as iron, in addition to antimicrobial effect of its own, it was chosen for a second trial of the experiment. Similar results were seen in examining the effect of copper ion alongside ciprofloxacin. While the lower concentration of copper (0.5 mg/L) did not have an effect on resistance development, the higher concentration (5 mg/L) increased ciprofloxacin resistance development in *E. coli* (Figure 2). As with iron, copper has been known to bind with ciprofloxacin resulting in reduced bioactivity, which is likely the cause of this increase in resistance. Longitudinal studies with iron and copper were repeated with doxycycline which had also displayed significant interaction with the two metal ions. Both copper and iron were found to significantly increase doxycycline resistance development toward doxycycline in *E. coli* (Figure 3). Unlike iron, copper has antimicrobial activity of its own which may have also contributed to the resistance development; however, at the low concentrations studied, copper activity against *E. coli* was minimal compared to the effect of the binding between copper and ciprofloxacin that was observed. In order to confirm the effect of chelation compared to the antimicrobial activity of copper, we chose to conduct an experiment with copper and streptomycin, as this combination previously did not show any interaction. These results showed similar resistance development between the conditions in the presence of copper and those with only streptomycin with no statistical difference between the conditions (Figure 4).

**Figure 5:**
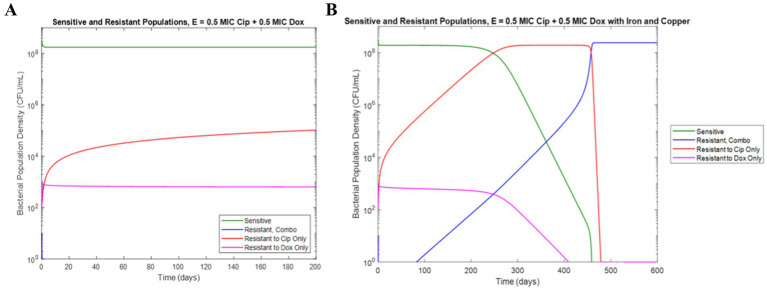
Simulation of resistance development under 0.5X MIC ciprofloxacin and 0.5X MIC doxycycline in the **(a)** absence of iron and copper **(b)** presence of 5 mg/L iron and 5 mg/L copper.

In order to probe the potential effects of these metal-antibiotic interactions on the development of AMR in wastewater environments, metal concentrations were added to our previously developed model of antibiotic resistance in continuous flow which simulates conditions more similar to real wastewater, and the outputs in the presence and absence of metal ion concentrations were compared. Previously, we developed and experimentally validated a model of the antagonistically interacting doxycycline and ciprofloxacin and we found that *E. coli* populations grown with these antibiotics showed an antibiotic ratio-dependent development of resistance (Sutradhar et al., 2023). Specifically, conditions with a lower MIC fraction of doxycycline compared to ciprofloxacin showed more resistance development than conditions with an equal ratio or greater relative doxycycline concentration (Sutradhar et al., 2023). After the addition of iron and copper concentrations to the model, we simulated the same conditions in the presence and absence of 5 mg/L iron and 5 mg/L copper. Interestingly, in the simulation of a combination of concentrations of 0.5X MIC ciprofloxacin and 0.5X MIC doxycycline, the addition of iron and copper ions predict the development of a *E. coli* population resistant to both antibiotics that was not seen in the absence of the metal ions (Figure 5).

## 4 Discussion

Overall, we found that metal ions, such as iron and copper, commonly found in wastewater, can significantly affect the development of antibiotic resistance for certain types of antibiotics. We observed significant interactions between these metal ions and both ciprofloxacin and doxycycline, leading to changes in the bioactivity of these clinically important antibiotics against *E. coli*. While it can be noted that Fe^3+^ ion has very low solubility in water— 10^−10^ M at pH of 7 (Supplementary Data) — increasing iron concentration was observed to decrease bioavailability of ciprofloxacin and doxycycline far beyond this point and (Table 3 and Figure 1). Thus, it is possible that the availability of iron for chelation extended beyond the concentration fully solubilized. Furthermore, we observed that the reduction in bioactivity from the presence of iron and copper increased the development of resistance to ciprofloxacin and doxycycline over time. These results are in agreement with previous literature on the chelation activity of these antibiotics on these metal ions and evidence that subinhibitory ciprofloxacin concentrations can lead to the development of ciprofloxacin resistance (Faure et al., 2021; Kara et al., 1991; Ghandour et al., 1992; Albert and Rees, 1956; Ching and Zaman, 2020). Additionally, for streptomycin, which did not have an observable interaction with either metal, resistance was shown to be unaffected by the presence of copper. Combined with the results of the other antibiotic-metal combinations studied, we can conclude that antibiotic chelation of metal ions can have a significant effect on AMR development over time. Though the effects of metal ions on the transfer of ARGs in wastewater has previously been noted, this mechanism of resistance development in response to metal ions has not previously been observed (Hubeny et al., 2021; Zhang et al., 2018); thus, these results present a novel finding not previously reported in studies of ciprofloxacin-metal or doxycycline-metal interactions. These effects were then incorporated into a computational model of AMR in wastewater. The model simulations predicted that metal-antibiotic interactions can significantly increase the development of antibiotic resistance over time in certain conditions. This finding is consistent with both the previously ratio-dependent behavior of the doxycycline-ciprofloxacin combination resistance development (Sutradhar et al., 2023) and the relatively high doxycycline metal ion chelation observed in this study. Thus, the increased resistance development predicted by model simulations can be attributed to increased chelation activity of doxycycline, activating the ratio-dependent resistance development behavior noted previously by tipping the antibiotic ratio toward a greater relative ciprofloxacin concentration. While the effects of metal ions on increased HGT of antibiotic resistance genes in the environment have been recorded, the effect of antibiotic-metal ions binding on resistance acquisition has not previously been modeled. Moreover, because other antibiotics in the classes of doxycycline and ciprofloxacin exhibit chelation with copper and iron (Faure et al., 2021; Kara et al., 1991; Ghandour et al., 1992; Albert and Rees, 1956), the model predictions may be applicable to tetracyclines and quinolones more broadly.

While our studies have demonstrated that metal-antibiotic interaction can significantly affect AMR development in wastewater, we note that our studies have limitations. One limitation was that we only studied a small number of metal-antibiotic combinations and as such cannot conclude that these effects will occur for metal-antibiotic interactions. Future studies investigating a wider array of trace metal ions could further illuminate the full impact of these ions on wastewater AMR. Further investigation of the other effects of metal ions on bacterial growth and resistance development, such as changes in pH and various organ-metal interactions could also give insight to the varied bacterial mechanisms affected by these pollutants. In order to further simulate wastewater conditions, future studies could also be conducted with more complex bacterial communities, lower temperatures, and media with closer nutrient composition to real wastewater. Additionally, we were limited by the infeasibility of experimentally validating our computational findings using continuous flow experimental setups due to the long timeframe and large resource requirements. Further innovation in experimental models of wastewater AMR will be required to fully understand the effects of metal-antibiotic interactions on AMR development.

## 5 Conclusion

In this study, we have demonstrated that metal ions alongside antibiotic residues in wastewater can significantly effect on the emergence of AMR over time. Through both experimental validation and computational modeling, we have observed that interaction between common metal ions and antibiotic residues can result in bioactivity changes leading to increased AMR development. Furthermore, because the effect of metal ions acts on the antibiotics, these findings have the potential for applicability beyond *E. coli* because altered bioactivity will have an effect on any bacteria with susceptibility to ciprofloxacin and doxycycline. These findings have important implications for understanding the effects of industrial runoff of metal ions in wastewater on AMR and highlight the need to consider the presence of metal ions when assessing the risk of AMR development in wastewater environments. For instance, the model may be able to determine acceptable metal ion levels for given environmental antibiotic levels in low resource settings where wastewater treatment options are limited. Thus, the developed model has potential to be used as a prediction tool for policy makers in both public health and environmental regulation in setting standards for acceptable metal ion and antibiotic residue levels in wastewater systems.

## Data Availability

The original contributions presented in the study are included in the article/Supplementary material, further inquiries can be directed to the corresponding author.
